# A gifted SNARC? Directional spatial–numerical associations in gifted children with high-level math skills do not differ from controls

**DOI:** 10.1007/s00426-020-01354-9

**Published:** 2020-05-24

**Authors:** Yunfeng He, Hans- Christoph Nuerk, Alexander Derksen, Jiannong Shi, Xinlin Zhou, Krzysztof Cipora

**Affiliations:** 1 Student Psychological Counseling Center, Liaoning University, Shenyang, China; 2 Department of Psychology, University of Tuebingen, Tuebingen, Germany; 3 LEAD Graduate School and Research Network, University of Tuebingen, Tuebingen, Germany; 4 Leibniz-Institut für Wissensmedien, Tuebingen, Germany; 5 CAS Key Laboratory of Behavioral Science, Institute of Psychology, Chinese Academy of Sciences, 16 Lincui Road, Chaoyang District, Beijing, 100101 China; 6 Department of Psychology, University of Chinese Academy of Sciences, Beijing, China; 7 Department of Learning and Philosophy, Aalborg University, Aalborg, Denmark; 8 State Key Laboratory of Cognitive Neuroscience and Learning and IDG/McGovern Institute for Brain Research, Beijing Normal University, Beijing, China; 9 Advanced Innovation Center for Future Education, Beijing Normal University, Beijing, China; 10 Siegler Center for Innovative Learning, Beijing Normal University, Beijing, China; 11 Centre for Mathematical Cognition, Loughborough University, Loughborough, LE 3TU UK

## Abstract

The SNARC (Spatial-Numerical Association of Response Codes) effect (i.e., a tendency to associate small/large magnitude numbers with the left/right hand side) is prevalent across the whole lifespan. Because the ability to relate numbers to space has been viewed as a cornerstone in the development of mathematical skills, the relationship between the SNARC effect and math skills has been frequently examined. The results remain largely inconsistent. Studies testing groups of people with very low or very high skill levels in math sometimes found relationships between SNARC and math skills. So far, however, studies testing such extreme math skills level groups were mostly investigating the SNARC effect in individuals revealing math difficulties. Groups with above average math skills remain understudied, especially in regard to children. Here, we investigate the SNARC effect in gifted children, as compared to normally developing children (overall *n* = 165). Frequentist and Bayesian analysis suggested that the groups did not differ from each other in the SNARC effect. These results are the first to provide evidence for the SNARC effect in a relatively large sample of gifted (and mathematically highly skilled) children. In sum, our study provides another piece of evidence for no direct link between the SNARC effect and mathematical ability in childhood.

**Electronic supplementary material:**

The online version of this article (10.1007/s00426-020-01354-9) contains supplementary material, which is available to authorized users.

## Introduction

### Spatial–numerical associations and the SNARC effect

From behavioural indexes to neural signatures, a large body of evidence shows bidirectional links between quantity/number processing and space (Cipora, Schroeder, Soltanlou, & Nuerk, 2018a; Dehaene, Bossini, & Giraux, 1993; Galton, 1880). This heterogeneous family of phenomena is referred to as *S*patial–*N*umerical *A*ssociations (SNAs, e.g., Fischer & Shaki, 2014; Patro, Nuerk, Cress, & Haman, 2014; Cipora et al., 2018a for a recent taxonomy). One of the most thoroughly studied SNAs, showing association between numerical magnitude and directions in space, is the *S*patial-*N*umerical *A*ssociation of *R*esponse *C*odes (SNARC) effect (Dehene, Bossini, & Giraux, 1993). It denotes the observation that in a timed bimanual setup, responses to relatively small/large magnitude numbers are faster on the left-/right-hand side, respectively. The SNARC effect can be observed in multiple tasks (e.g., parity judgment, magnitude classification, and phoneme monitoring). It is present irrespective of number presentation format (e.g., Arabic, number words, auditory numbers, dice patterns, nonsymbolic numbers, and numbers presented in a tactile format) and response mode (bi- and uni-manual, pointing, bipedal, and eye movements; see Nemeh, Humberstone, Yates, & Reeve, 2018; Patro, & Shaki, 2016a, b for effects in non-symbolic numbers; Wood, Willmes, Nuerk, & Fischer, 2008 for a meta-analysis; Fischer & Shaki, 2014 for a review).

SNARC-like effects can be observed very early in development, including in neonates (see Di Giorgio et al., 2019; de Hevia, Veggiotti, Streri, & Bonn, 2017) and prevail in subsequent stages of development. For instance, preliterate kindergarten children, already show non-symbolic SNARC effects (Patro & Haman, 2012; for possible mechanisms, see Nuerk et al., 2015). When children enter primary school, and develop literacy and familiarity with symbolic numbers, their SNARC effects can be measured through tasks typically used with adults. At the age of about 7 years, the SNARC effect can be observed in a symbolic magnitude judgment task (Galen & van Reitsma, 2008) and in a parity judgment task at the age of about 9 years (Berch et al., 1999). The SNARC effect in early adolescents (fifth- and sixth-graders, mean age approximately 11 years old) has been documented in a large-scale (*n* = 429) study by Schneider, Grabner and Paetsch (2009). The SNARC effect can be also observed in adult participants of various ages (Hoffmann, Mussolin, Martin, & Schiltz, 2014a, b; see also Ninaus et al., 2017 for a cross-sectional study; Wood et al, 2008, for a meta-analysis).

Despite being a well-established and easily replicable phenomenon (see Cipora, Soltanlou, Reips, & Nuerk, 2019a for a large-scale online replication), the underlying mechanisms of the SNARC effect are still a subject of debate (e.g., Dehaene et al., 1993, but van Dijck & Fias, 2011, Schroeder, Nuerk, & Plewnia, 2017 for opposing views). The determinants of left-to-right directionality are also debated and opposing views emphasize the role of innate biases (e.g., Rugani, Regolin & Vallortigara, 2010) or cultural factors such as dominant reading/writing direction and other implicit spatial biases in a society (e.g., Patro, Fischer, Nuerk, & Cress, 2016a, b; Patro, Nuerk, & Cress, 2016; Shaki, Fischer & Petrusic, 2009). Interestingly, as typically quantified, the SNARC effect can be observed in about 70–80% of individuals (e.g., Wood et al., 2008; Cipora et al., 2016, Cipora et al., 2019b). Since individual differences can be observed, another vital question in the debate is: which variables correlate with the SNARC effect?

Some correlates of the SNARC effect have been repeatedly reported in the literature. For instance, reaction time (RT) characteristics in a task measuring the SNARC effect are related to the SNARC effect itself: slower and more varied responses, longer mean RT, and larger intraindividual variability in RT, (SD)RT are linked to a stronger SNARC effect (Cipora & Nuerk, 2013; Gevers, Verguts, Reynvoet, Caessens, & Fias, 2006; Wood et al., 2008, for a meta-analysis). Therefore, it is possible to find a consistent pattern of correlations between the SNARC effect and other measures (especially when large samples are tested and reliable tasks are utilized (e.g., Cipora et al., 2019a). On the other hand, it is still unknown if and how the SNARC effect correlates with other constructs, especially with math skills level.

### The relationship of the SNARC effect and math skill: does its direction depend on age?

Math skills can be considered a natural candidate to be a correlate of the SNARC effect: elementary spatial mapping of numbers might be related to the efficiency of more advanced number processing such as arithmetic (see Cipora, He, & Nuerk, 2020b). Note that similar discussions on whether high/low cognitive ability in a specific domain modulates other processes and representations are present in other domains of cognitive psychology as well, and similar to this discussion, they also do not bring very consistent results. For instance, there is a long-lasting debate on whether bilingualism influences efficiency of cognitive control processes (e.g., De Bruin et al., 2015; Paap et al., 2015), or whether physical exercise modulates cognitive processes such as perception or attention (e.g., Mann et al., 2007).^1^

As regards the SNARC effect, most studies to date, considering both children and adults, have not found such a relationship (see Cipora et al., 2018b for a review considering different ways to quantify math/arithmetic skill used across studies). Specifically, to the best of our knowledge, there are eleven published studies investigating the relationship between the SNARC effect and math skills in adults (review by Cipora et al., 2018b does not consider three adult studies; Cipora et al., 2019a, Kramer et al., 2018 and Toomarian, Meng, & Hubbard, 2019, which were published afterwards; see also Table 1 in Cipora et al., 2020b for a complete overview). Out of these, eight studies (Dehaene et al., 1993, Exp. 1; Fischer & Rottmann, 2005; Bonato et al., 2007, Exp. 1; Bull et al., 2013, Exp. 2; Cipora & Nuerk, 2013; Goebel et al., 2015; Cipora, et al., 2019a, Toomarian et al., 2019) reported null results. The other three studies (Hoffmann et al., 2014a, b; Cipora et al., 2016; Kramer et al., 2018) reported that individuals characterized as having better math skills had a weaker SNARC effect. In the case of child studies, there are seven published studies investigating the relationship between math skills and the SNARC effect. Out of these, in three studies (Schneider et al., 2009, Exp. 2; Crollen & Noel, 2015; Gibson & Maurer, 2016) no such effect was found. In the remaining four studies (Bachot et al. 2005; Georges et al., 2017; Crollen et al., 2015; Hoffmann et al., 2013), children characterized as having a higher level of math skills had a stronger SNARC effect, and the SNARC effect was not present in children who experienced math difficulties. Of note, even in the case of studies in which a significant relationship was found, the observed effect sizes were either small or moderate (only in one case did the correlation exceed 0.30; in the case of group comparisons, corresponding effect sizes were also small, see Cipora et al., 2018b). Thus, our reading of the literature is that for the relationship between the SNARC effect and math skill, there probably has been a null and possibly small effect size in children, but certainly not a medium or large one.

**Table 1:** Descriptive information of participants

		Overall *n *(female)	Grade	*n *(female)	Mean age (years)	Age range (years)
Main analysis	Gifted children	74 (32)	3	26 (8)	9.45 ± 0.297	8.92–9.83
			4	29 (17)	10.31 ± 0.301	9.75–10.75
			5	19 (7)	11.60 ± 0.252	11.00–12.00
	Normal children	91 (41)	3	31 (15)	9.50 ± 0.427	9.00–11.33
			4	26 (8)	10.15 ± 0.331	9.58–11.08
			5	34 (18)	11.44 ± 0.395	10.67–12.58
Intelligence- based analysis	Intellectually gifted children	44 (18)	3	22 (7)	9.41 ± 0.295	8.92–9.83
			4	17 (10)	10.34 ± 0.311	9.75–10.75
			5	5 (1)	11.50 ± 0.295	11.00–11.75
	Intellectually normal children	17 (9)	3	6 (3)	9.43 ± 0.244	9.08–9.83
			4	3 (1)	10.36 ± 0.337	10.00– 10.67
			5	8(5)	11.36 ± 0.336	10.67–11.83
Arithmetic performance-based analysis	Arithmetically gifted children	13 (2)	3	6 (0)	9.21 ± 0.246	8.92–9.58
			4	6 (2)	10.13 ± 0.311	9.75–10.67
			5	1 (0)	11.50 ± 0	11.50–11.50
	Arithmetically normal children	47 (23)	3	14 (8)	9.57 ± 0.574	9.00–11.33
			4	15 (5)	10.18 ± 0.287	9.58–10.67
			5	18 (10)	11.46 ± 0.408	10.67–12.58

In regard to adults, out of the three studies which reported a significant relationship between the SNARC effect and math skills two considered extreme groups: individuals with math difficulties, who turned out to reveal stronger SNARC effect than other groups (Hoffmann et al., 2014a, b) and professional mathematicians, who did not reveal the SNARC effect (Cipora et al., 2016). These extreme groups mostly drove the observed effects in these studies. In the case of individuals with math difficulties, the explanation provided by the authors is that in these participants the retrieval of the parity of a given number was related with higher executive function load. This consequently lead to less efficient inhibition of the task-irrelevant spatial representation, and amplified the observed SNARC effect (Hoffmann et al., 2014a, b). The lack of the SNARC effect in professional mathematicians (and a difference when compared to individuals with normal math skills level) was attributed to more abstract number processing or more flexible spatial-numerical representations (Cipora et al., 2016).

While for adults, extreme groups from both sides of the spectrum (high and low skills) have been tested, that has not been the case for child studies so far. The four child studies conducted, which showed the relationship between the SNARC effect and math skill, considered either typically developing children with typical levels of skill in math (e.g., Georges et al., 2017; Hoffmann et al., 2013), or children with developmental disorders and/or math problems (e.g., Bachot et al., 2005; Crollen et al., 2015). Authors of these latter studies interpret their results in terms of decreased saliency of left to right mapping of numbers in children with non-verbal learning disabilities (Crollen et al., 2015)/visuospatial disabilities (Bachot et al., 2005), role of spatial numerical associations for math skills at early stages of math development (Georges et al., 2017), or greater familiarity with Arabic numbers being related to stronger SNARC (Hoffmann et al., 2013). Importantly, none of the studies considered children highly skilled in math.

Obviously, there are no professional mathematicians among children, but we can examine highly intellectually gifted children, who typically excel in math as well (see, e.g., Primi, Ferrão, & Almeida, 2010; Roth, Becker, Romeyke, et al., 2015)^2^ and receive more intense math training as compared to their peers. Therefore, focusing on gifted children can provide complimentary evidence to the debate on the relationship between the SNARC effect and math skills. Testing this understudied group can reveal whether the relationship with the SNARC effect in children is linear (i.e., gifted children reveal even stronger SNARC than peers with typical math skills levels, end the mathematically challenged children experience the weakest/none SNARC) or non-linear (i.e., both mathematically highly skilled and mathematically challenged children with do not show the SNARC effect, but due to different mechanisms). In the case of children with math difficulties, it could be due to non-efficient, non-automatized number processing, while for skilled/gifted children it could originate from flexible representations (see Moeller et al., 2011, Fig. 3, for a similar non-monotonic suggestion as concerns the distance effect). Apart from providing additional evidence for the relationship between the SNARC effect and math skill, testing highly gifted children may potentially be free from confounding factors related to testing atypically developing children. Specifically, mathematically challenged children can be characterized with longer and more variable reaction times, and as we already mentioned these RT parameters influence the SNARC effect.

To sum up, there is diverging evidence for the relationship between the SNARC effect and math skills. These diverging results cannot be accounted for by differences in operationalization of the math skills either. However, if such a relationship did exist, its direction seems to differ between children and adults. In adults, the SNARC effect has tended to be weaker in highly skilled groups and stronger in groups with lesser skill. In children, the effects tended to be weaker in groups with lesser skill. However, in the case of child studies, the SNARC effect was not investigated in groups with high level math skills, and as mentioned above, there may be some confounds in measuring the SNARC effect in children with math difficulties. As giftedness and math skills are tightly related, testing gifted children can fill an obvious gap in the existing evidence on the relationship between the SNARC effect and math skills during lifetime development.

### The current study

The current study aims to investigate the SNARC effect in gifted and normal children. To the best of our knowledge, this is the first attempt at exploring the SNARC effect in such a group with a relatively large sample of children that is compared to an age matched control group.

First, we aim to replicate the SNARC effect in our sample. We expect to observe the SNARC effect, at least in the control group. This will be the starting point for following analyses.

Second, we wish to investigate the SNARC effect in the group of highly gifted children. As we have already discussed, based on the existing literature it is hard to come up with a direct prediction regarding the SNARC effect in the gifted children. Specifically, there is some evidence favouring all three of the following theoretically possible scenarios:

1. Gifted children do not differ from controls in their SNARC effect. As documented in several child and adult studies, the SNARC effect can be independent of math skills level. For this reason, it is possible that the two groups will not differ in the SNARC effect.
2. Gifted children have a stronger SNARC effect than controls. In most of the studies in which the relationship between the SNARC effect and math skills was found, groups characterized by extreme levels of math skills were tested. Highly gifted children are thus a group, in which one might expect such an effect. In keeping with other studies testing children of this age, one might expect gifted children to reveal a stronger SNARC effect than the controls.
3. Gifted children have a weaker SNARC effect than the controls. As described above, there are reasons to believe that gifted children would differ from controls. However, as the studies to this date did not test children with a very high level of skill in math, it may be also possible that this group has a similar pattern to professional mathematicians (i.e., weaker or non-existent SNARC effect), and the relationship between the SNARC effect and math skills in children is not linear.

## Methods

### Participants

A total of 182 children (from six different classrooms) participated in this study. They were recruited from Grades 3 to 5, at the end of their second semester (July), in a gifted education school in Beijing. In this school there are separate classes for gifted and normal pupils. Children following normal curriculum were considered as a control group. Out of these, 17 participants had to be excluded from analyses: two of them due non-completion of both blocks of the parity judgment task and 15 for whom we did not have a valid reaction time within at least one cell (i.e., number × response side configuration). Thus, analyses were conducted on 165 children (cf. Table 1, upper part). There were no significant differences between gifted children and normal children in age, *t* (163) =  − 0.495, *p* = 0.621; or in gender, *χ*^*2*^ (163) = 0.054, *p* = 0.816. Children for whom data on the Raven’s Standard Progressive Matrices (*n* = 10) and arithmetic task (*n* = 7) was missing were not included in analyses considering these measures.

Gifted children to be enrolled in a dedicated curriculum are selected every year, according to multiple criteria and methods, from about 3000 candidates recruited from a region populated by about 20 million people (Shi & Xu, 2004; Shi & Zha, 2000). The selection takes place before children enter the first grade. The main selection steps include an application, a primary screening test (several classical intelligence tests), a second test (assessment of cognitive abilities, personality traits and creativity), and behavioural observation in a gifted educational environment. The children’s physical condition and learning abilities are also confirmed. The children enrolled into the gifted curriculum were within the top 5% of all the candidates. This screening process has been implemented for almost 40 years and it has turned out to be effective (Liu et al., 2007; Shi et al., 2013).

All participants had normal or corrected-to-normal vision. Prior to participation, written informed consent was collected from both the parents and children. The study was approved by the Ethics Committee of the Institute of Psychology, Chinese Academy of Sciences.

### Experimental materials

#### Intelligence test

Fluid intelligence of the participants was measured with Raven’s Standard Progressive Matrices (RSPM; Raven, Court, & Raven, 2004). The test was administered in the standard paper-and-pencil format with a time limit of 45 min. In the correlation analyses, raw scores (theoretical range 0–60) were used. Normalized scores used for subgroup selection were calculated according to Chinese norms (Zhang & Wang, 1989).

#### Parity judgment

A classic bimanual parity judgment task was used. Participants were to assess whether a number presented on a screen (front size 100px, presented in black centrally against a white background) was odd or even with a key press. Single-digit Arabic numbers from 0 to 9 were used. Numbers were presented until the participant’s response, and the next trial followed immediately. Presentation order was randomized.

There were two experimental blocks (order fixed across participants). They were separated by another task (see “Procedure” section below). In the first block, participants pressed the Q key on a standard computer keyboard (i.e., left side response) for odd numbers and the P key (i.e., right side response) for even numbers. Response-to-key assignment was flipped in the second block. Within each block, each number was presented four times. Therefore, the total number of trials was 80. Experimental blocks were preceded by a practice session consisting of 20 trials. If a child reported difficulties in understanding the task during the practice session, any questions were answered, and the practice session was repeated. The instruction stressed the importance of both the speed and accuracy of responses. The entire task together with instructions and practice sessions took about five minutes to complete.

According to previous Chinese studies on the development of the SNARC effect (Liu et al., 2018; Yang et al., 2014), Chinese children as young as kindergarten age have a clear understanding of parity. We have also confirmed our participants’ understanding of the parity concept with their teachers prior to the experiment and double-checked with the participants during and after experiments.

#### Arithmetic task

It was not possible to use the same measures as previous studies, in particular those, which reported significant effects: Hoffmann et al. (2013) tested kindergarteners (at this age no selection to gifted curricula is made in China), and measured proficiency with Arabic numbers as a measure of math skills (which would be too easy for our sample of 3–5 graders). Bachot et al. (2005) and Crollen et al. (2015) tested children in much wider age range than we did (7–12 and 6–13 respectively) and their participants had a diagnosis of learning disabilities. Bachot et al. (2005) measured math skills with arithmetic task (complex addition), number concepts, and simple automatized number facts. Crollen et al. (2015) used basic numerical reasoning. In our opinion, these tasks are not suitable for testing gifted children, because these tasks designed for children with learning abilities are likely leading to ceiling effects in our sample. Georges et al. (2017) used standardized math test (Heidelberg Mathematics Test—HRT) comprising mental additions, subtractions, multiplications, divisions, number equations filling and number comparison. All tasks are timed. However, HRT is designed to detect dyscalculic children; it can be used in the normal range, but does not differentiate so well for highly gifted children. Nevertheless, our approach resembles that of Georges et al. (2017). We used standardized (and normalized) timed arithmetic task proven to be effective in Chinese cultural and educational context. For this reason, we believe that this task is suited to answer a general question we ask, that is whether the SNARC effect relates to math skills in highly gifted children. In the current study, arithmetic ability was assessed using a two-digit by one-digit number multiplication task (see He et al., 2016; Wei et al., 2012a, b). Participants were presented with a problem (e.g., 32 × 3), and four response alternatives were displayed below. Two alternatives were corresponding to the Q and the other two to the P key. All response alternatives had the same numbers of units, but a different number of decades (e.g., 66, 96, 56, 86). Response alternatives ranged from 10 to 90. Participants were to press Q/P if the correct answer was one of those on the left/right side of the display. There were 76 problems in total. Participants were to solve as many problems as possible within a time limit of 2 min. The importance of both speed and accuracy were stressed in the instruction, use of calculation aids (e.g., paper and pencil) was forbidden.

As the correct answer is selected from four alternatives, and the response collected from the participant indicates two out of four alternatives, calculating the overall score considers a correction for guessing as suggested by Guilford. Specifically, the formula $S =R-W/(N-1)$ is used, where *R* is the number of correct responses, *W* is the number of incorrect responses, and *N* is the number of alternative responses to each item. In our case, as *N* = 2, the formula simplifies to $R-W$.

The resulting score *S* (theoretical range − 76 to 76) is defined as the number of items that the participant can actually answer without guessing (Guilford, 1936; Cirino, 2011). Normative data for this task were obtained from a previous study on 1556 primary school children in the greater Beijing area of China, including the mountain area, suburbs and urban area (Wei et al., 2012a, b).

The parity judgment task and arithmetic task were implemented in a web-based application “Online Psychological Experiment System (OPES)” (https://www.dweipsy.com/lattice).

### Procedure

This experiment was a part of a larger study on number processing of gifted children in comparison to children receiving a normal education. It comprised multiple sessions conducted in a group setup. In the first session, RSPM were administered in the familiar environment of the students’ classrooms, with paper and pen. The parity judgment task and arithmetic task were administered to each class in a computer classroom. The session in the computer room lasted 45 min, during which several computerized tasks were administered. The task order was as follows: (1) choice RT; (2) non-symbolic comparison (version 1); (3) parity judgment block 1; (4) estimate arithmetic; (5) numerical Stroop task (version 1); (6) non-symbolic comparison (version 2); (7) parity judgment block 2; (8) exact arithmetic; (9) numerical Stroop task (version 2). Here we only consider RSPM, the parity judgment task and the exact arithmetic task. Both sessions were administered within two weeks.

### Data preparation—quantifying the SNARC effect

Despite having used the full range of single digit numbers (0–9), in the main analysis, we excluded numbers 0 and 5. First, the number zero was shown to have a specific status (see Brysbaert, 1995; Fias, 2001; Armstrong, Gleitman, & Gleitman, 1983; Nuerk et al., 2004), and for number 5, which is in the middle of the range, we do not have any specific predictions in regards to its spatial associations. This stimuli set is considered the most typical in the SNARC effect literature (e.g., Georges et al., 2017). However, to give a full overview, the analysis considering the full number range is also reported.

Only correctly solved trials were considered for further analysis. The RT was the main dependent variable. Trials with RTs shorter than 200 ms were treated as anticipations and excluded from further analysis. The sequential trimming method (see Cipora & Nuerk, 2013) was applied: RTs ± 3SD outside the participant’s mean RT were removed sequentially. The proportions of trials considered in the analysis are summarized in Table 2.

**Table 2:** The descriptive statistics of all tasks

Group	Parity task				Arithmetic performance	Intelligence (RSPM)
	Accuracy^a^	Trials included^a^	Mean RT (SD) [ms]^a^	SD within participants, SD (RT) [ms]^a^	Mean (SD)	Mean (SD)
Overall	0.93/0.92	0.89/0.88	765 (165)/761 (156)	188/181	26.82 (6.72)	47.08 (6.16)
Gifted	0.93/0.93	0.89/0.89	698 (108)/698 (107)	150/151	28.55 (6.79)	49.81 (5.38)
Control	0.92/0.92	0.88/0.87	820 (182)/811 (171)	219/205	25.30 (6.32)	44.58 (5.78)

^a^Results without 0 and 5/with 0 and 5

Calculation of the SNARC effect followed the approach adapted byCipora et al. (2019a; see also Fias et al., 1996, Nuerk, Bauer, Krummenacher, Heller, & Willmes, 2005a, b). Specifically, for each participant a dRT (RT difference RH – LH) was calculated for each number. Subsequently, dRTs were regressed on number magnitude and contrast-coded parity (− 0.5 for odd and 0.5 for even numbers).^3^ Here we focus on the SNARC effect. The MARC effect (i.e., the parity contrast slopes; see Nuerk et al., 2004) is reported in Supplementary Material 1. As the sample was relatively large, it was also possible to investigate gender differences in the SNARC and MARC effects. These analyses are presented in Supplementary Material 2.

Following Cipora et al. (2019a) we considered both unstandardized (henceforth SNARC) and standardized (ST-SNARC) slopes. The latter were Fisher-Z transformed to approximate the normal distribution. Increasingly negative slopes correspond to a stronger SNARC effect.

### Data analysis

First, we tested for the presence of the SNARC effect at the whole sample level and in each group separately. Additionally, we tested for reliability of the SNARC slopes using the split-half method and adjusting for test length using the Spearman-Brown formula (see Cipora & Nuerk, 2013, see also Cipora et al., 2019b for a detailed description of the algorithm). In the next step, SNARC slopes were correlated with performance measures (mean and intraindividual variability of reaction times) in the parity judgment task, as well as with RSPM scores and performance in the arithmetic task.

Subsequently, the groups were compared. To obtain the largest power, we based our main group comparison on the Chinese selection process for placement in the gifted or normal curriculum (Main analysis). As one might question the Chinese selection system, we conducted two additional analyses considering subgroups selected based on additional criteria in order to check the robustness of our results: (1) *Intelligence-based analysis* children enrolled in the gifted curriculum who scored within the top 5% according to Chinese norms in RSPM (*n* = 44) were compared to children enrolled in the normal curriculum, whose scores fell within the 25th–75th percentile (*n* = 19); (2) *Arithmetic performance-based analysis* we compared pupils enrolled in the gifted curriculum, who scored within the top 5% in the arithmetic task (*n* = 13), to pupils enrolled in the normal curriculum, whose scores fell within 25th and 75th percentile (*n* = 47). Detailed information on participants considered in each of the analyses is presented in Table 1.

For group comparisons and correlations, we used frequentist correlations and *t *tests along with their Bayesian equivalents. BF_01_ are reported in the text; therefore, values above 1 show that the null hypothesis model is favoured over the alternative hypothesis model, and consequently, values < 1 indicate that alternative hypothesis model is favoured over the null hypothesis model. Typically, values > 3 and < 0.3 are considered as conclusive evidence. Nevertheless, there are no strict cutoff criteria, instead BF values can and should be treated in a continuous way (see Wagenmakers et al., 2018). Importantly, using Bayesian statistics allows us to provide positive evidence for the null hypothesis (i.e., lack of between group differences).

## Results

### Descriptive statistics

Descriptive statistics of all measures are presented in Table 2. Gifted children responded faster than controls in the parity judgment task, *t* (163) = − 4.97, *p* < 0.001, *d* = − 0.78, and their intraindividual variability in reaction times was smaller, *t* (163) = − 4.00, *p* < 0.001, *d* = − 0.63.^4^ They also achieved higher scores in RSPM, *t* (153) = 5.82, *p* < 0.001, *d* = 0.94, and on the arithmetic task, *t* (156) = 3.12, *p* = 0.002, *d* = 0.50.

### The SNARC effect

A robust SNARC effect was observed at the whole sample level (cf. Table 3). As expected, it was present in the control group. Crucially, it was also robust in the gifted group. This observation holds irrespective of which set of numbers was considered, and of whether unstandardized or standardized slopes were considered. In all cases, both the frequentist and Bayesian evidence are conclusive. To sum up, a robust SNARC effect was observed in all instances. Proportions of participants revealing negative slopes were comparable to those reported in the literature. On the other hand, reliabilities of the slopes were very low (cf. Table 3), which might be a problem for correlations of individuals, but is typically less of a problem for mean group differences in high N groups as follows from the central limit theorem.

**Table 3:** The summary of the SNARC effect: overall, within groups, and between group comparisons

Task property				SNARC		ST-SNARC	
				1–4 6–9	0–9	1–4 6–9	0–9
Reliability				0.13	0.17	0.18	0.31
SD slope				21.95	19.19	0.43	0.71
% neg. slopes				0.67	0.70	0.67	0.70
Slope	Overall	Mean		− 7.76	− 7.29	– 0.18	– 0.19
		*t* test against 0 (*df* = 164)	*t*	− 4.54	− 4.88	– 5.48	– 6.11
			*p*	< 0.001	< 0.001	< 0.001	< 0.001
			*d*	0.35	0.38	0.43	0.48
			BF_01_	8.177*e* − 4	2.038*e* − 4	1.468*e* − 5	7.408*e* − 7
	Gifted	Mean		– 5.23	− 5.79	– 0.17	– 0.18
		*t* test against 0 (*df* = 73)	*t*	– 2.32	− 3.12	– 3.09	– 3.73
			*p*	0.023	0.003	0.003	< 0.001
			*d*	0.27	0.36	0.36	0.43
			BF_01_	0.636	0.094	0.102	0.017
	Control	Mean		– 9.82	– 8.51	– 0.19	– 0.20
		*t* test against 0 (*df* = 90)	*t*	– 3.95	– 3.78	– 4.78	– 4.87
			*p*	< 0.001	< 0.001	< 0.001	< 0.001
			*d*	0.41	0.40	0.50	0.51
			BF_01_	0.008	0.014	4.286e − 4	3.061e − 4
	Group comparison	*t* test (*df* = 163)	*t*	1.34	0.30	0.24	0.30
			*p*	0.182	0.766	0.811	0.766
			*d*	0.21	0.05	0.03	0.05
			BF_01_	2.585	5.675	5.760	5.675

### The SNARC effect correlations

Correlations of all measures we considered are presented in Table 4. Expectedly, all SNARC measures are highly correlated. On the other hand, none of the SNARC measures correlated with RT characteristics in the parity judgment task. Expectedly, RT characteristics correlated very highly with each other. RT characteristics, RSPM, and arithmetic scores were correlated, which also could have been expected.

**Table 4:** Correlations between all measures considered in the study

Variable	(1)	(2)	(3)	(4)	(5)	(6)	(7)	(8)
(1) SNARC (1–4 6–9)	–	< 0.001	< 0.001	< 0.001	9.856	8.621	1.448	5.667
(2) ST-SNARC (1–4 6–9)	0.83^***^(0.78; 0.87)	–	< 0.001	< 0.001	4.186	2.526	6.350	9.194
(3) SNARC (0–9)	0.86^***^(0.81; 0.90)	0.71^***^(0.62; 0.78)	–	< 0.001	10.166	8.188	3.954	2.699
(4) ST-SNARC (0–9)	0.72^***^(0.63; 0.78)	0.80^***^(0.74; 0.85)	0.87^***^(0.83; 0.90)	—	6.177	1.877	8.585	7.363
(5) Mean RT	0.02 (− 0.13; 0.18)	0.11 (– 0.05; 0.25)	0.01 (– 0.14; 0.16)	0.08 (– 0.07; 0.23)	–	< 0.001	0.001	< 0.001
(6) SD (RT)	0.047(− 0.11; 0.20)	0.13 (– 0.02; 0.28)	0.05 (– 0.10; 0.20)	0.14 (– 0.01; 0.29)	0.88^***^(0.84; 0.91)	–	0.023	< 0.001
(7) RSPM score	0.16^*^(0.00; 0.31)	0.08 (– 0.08; 0.23)	0.11 (– 0.05; 0.26)	0.04 (– 0.11; 0.20)	– 0.31^***^(– 0.45; – 0.16)	– 0.28^***^(– 0.42; – 0.13)	–	< 0.001
(8) Arithmetic score	0.09(− 0.07; 0.24)	0.03 (– 0.12; 0.19)	0.13 (– 0.03; 0.28)	0.06 (– 0.09; 0.22)	– 0.39^***^(– 0.52; – 0.25)	– 0.37^***^(– 0.50; – 0.23)	0.35^***^(0.20; 0.48)	–

Below the diagonal: Pearson correlations (95% CI); *p < 0.05, **p < 0.01, ***p < 0.001; Above the diagonal: BF_01_; SNARC – unstandardized SNARC slope; ST-SNARC – standardized SNARC slope [both calculated with numbers 0 and 5 being excluded (1–4 6–9) and with all numbers included (0–9)], MeanRT – mean RT in the parity judgment task, SD(RT) – standard deviation of RT within a participant, RSPM – raw score in Raven Standard Progressive Matrices, Arithmetic – raw score in the arithmetic task.

On the other hand, the SNARC effect measurements were not correlated with arithmetic performance scores. Bayesian analyses provided support for lack of correlations, only in one case could the Bayesian evidence be considered inconclusive (BF_01_ < 3), but the analysis still favoured the null hypothesis model.

In the case of a relationship between the SNARC effect measures and the RSPM, only one correlation reached significance, however this correlation was very low, and the Bayesian analysis still favoured the null hypothesis model (despite being largely inconclusive).

To sum up, the correlational analysis did not provide evidence that the SNARC effect is related to math skills nor intelligence. Moreover, Bayesian analyses supported the null hypothesis models.

### Between-group comparisons

#### Main analysis

The main between-group comparisons (cf. Bottom part of Table 3) did not show any difference between gifted children and the control group in respect to the SNARC effect measures.^5^ In all but one of the cases, the Bayesian analysis favoured the null hypothesis model (only in one case the evidence was inconclusive). The conclusion that gifted children do not differ from controls is also supported by two subsequent analyses considering more conservative group allocation (see “Data analysis” section).

#### Intelligence-based analysis

In the comparison considering the additional criterion of RSPM scores, there were no between group differences in any of the SNARC effect measures: the SNARC effect without 0 and 5, *t* (59) = 1.60, *p* = 0.116, *d* = 0.46, BF_01_ = 1.244; the ST-SNARC effect without 0 and 5, *t* (59) = 0.57, *p* = 0.573, *d* = 0.16, BF_01_ = 3.074; the SNARC effect with 0 to 9, *t* (59) = 0.82, *p* = 0.413, *d* = 0.24, BF_01_ = 2.668; the ST-SNARC effect with 0 to 9, *t* (59) = 0.17, *p* = 0.864, *d* = 0.05, BF_01_ = 3.469.^6^

#### Arithmetic performance-based analysis

In the comparison considering additional criterion of arithmetic performance there was no between group difference either: the SNARC effect without 0 and 5, *t* (58) = 0.85, *p* = 0.402, *d* = 0.27, BF_01_ = 2.449; the ST-SNARC effect without 0 and 5, *t* (58) = 0.69, *p* = 0.495, *d* = 0.22, BF_01_ = 2.700; the SNARC effect with 0 to 9, *t* (58) = 0.90, *p* = 0.373, *d* = 0.28, BF_01_ = 2.366; the ST-SNARC effect with 0 to 9, *t* (58) = 0.90, *p* = 0.374, *d* = 0.28, BF_01_ = 2.366.^7^

## Discussion

### Overview

In the current study we explored the SNARC effect in gifted children in comparison with a control group. We found a robust SNARC effect in both groups. As expected, groups differed considerably in performance in RSPM and arithmetic performance, with the gifted group performing better. In the gifted group the RTs were also shorter and less variable, which was to be expected as RTs are related to fluid intelligence (for reviews, Neisser et al., 1996; Vernon, 1987). All together these results further validate group division originally based on the Chinese system for selection of gifted children. Nevertheless, groups did not differ in any measure of the SNARC effect, no matter which additional criterion was used to make group distinction even clearer. Importantly, the lack of between group differences in the SNARC effect cannot be accounted for by low reliability of the SNARC effect in our study. As low reliability can be problematic for correlational analyses, it is not so in the case of between group comparison. Specifically, reliability was low at the individual level. That is, the individual estimates of slope might have been affected by measurement error. Nevertheless (in line with fundamental assumptions of classical test theory), the error was randomly distributed, therefore, in case of relatively large samples (groups) it should cancel out in case of the between group comparison. For this reason, the group comparison remains meaningful and interpretable.

We have also observed that the SNARC effect was not related to continuous measures of arithmetic performance nor intelligence. These results should be interpreted with caution due to low reliability. Nevertheless, they point to the same direction (no SNARC – math skills relation) as the between group comparisons. Contrary to results reported in the literature, the SNARC effect did not correlate with reaction time characteristics in our Chinese samples.

Importantly, in virtually all cases conclusions from frequentist and Bayesian analyses converge to favour null hypothesis models (i.e., no correlation/no between group differences). Additionally, the SNARC effect did not change significantly with age or grade. On one hand, it may be because the age range of our participants was relatively small (but see Berch et al., 1999 for contrasting results showing differences in SNARC between grades). On the other hand, our observation is in line with previous Chinese studies on the development of the SNARC effect (from kindergarteners to sixth graders, Yang et al., 2014 and from second graders to adults, Liu et al., 2018).

### SNARC in gifted children–reasons for a difference

Studies reporting relations between math skills and the SNARC effect have proposed some explanations as to why such a relationship was observed. These interpretations refer to (1) differences in automatic magnitude processing, and (2) representation abstractness. This idea was recently elaborated in a model framework by Cipora et al. (2020b). The model suggests multiple mechanisms on why the SNARC effect should or should not be related to math skills. Please note that this study was not aimed at verifying such a model: it was conducted before the actual model was developed. Thus, the below considerations are post-hoc explanations of null results observed here, and should be treated as such.

#### Automatic magnitude processing

As proposed by Hoffmann et al. (2013) the difference in the SNARC effect depending on math skills level might be related to differences in automatic number processing in children. This is a very plausible explanation, because automatic processing of magnitude (especially in a magnitude-irrelevant parity judgment task) is a prerequisite for magnitude-related effects to occur. However, in the case of our participants, we can assume that they all reached a sufficient level of such processing. First, our participants were older than those tested in the study by Hoffmann. Second, there is empirical evidence showing automatic magnitude processing in Chinese children of this age (Yao et al., 2015). Eventually, as it was shown in multiple international studies, Chinese children are ahead of their Western peers when it comes to early math skills (e.g., Cvencek, Nasir, O'connor, Wischnia, & Meltzoff, 2015). Moreover, it needs to be mentioned that apart from cross-cultural differences, the entire sample tested in the present study (including the control group), was at above-average level compared to Chinese children of this age in regard to math skills.

Importantly, in most of the studies which reported a relationship between math skills and the SNARC effect at the age of our participants, tested samples of **individuals who had math difficulties/developmental disorders (Bachot et al., 2005; Crollen et al., 2015). Only one study (Georges, Hoffmann, & Schiltz, 2017) found a relationship between the SNARC effect and math skills level in typically developing children of the age of our participants. It is thus possible that at least in some of these studies the relationship between math skills and the SNARC effect was driven by differences in automaticity of magnitude processing. Such differences might not have occurred in our sample. On the contrary, it is likely that our participants have already reached the necessary level with automatic magnitude processing, and it did not differ between the groups to an extent which could cause differences in the SNARC effect. This can explain why gifted children did not reveal a stronger SNARC than their peers from the control group (i.e., the effect reported in some child studies on the SNARC and math skills). Potentially, one could use the interference measure in the physical size condition as a measure of automatic magnitude processing of numbers (numerical magnitude affects the decisions about physical size of presented digits). However, at the same time, the observed effect is not only measuring automatic number magnitude processing, but also the efficiency of inhibition processes (see Cipora et al., 2020b). On the one hand, we could expect the gifted children to process magnitude automatically (assuming there are still any differences in that respect), but also we would expect them to have more efficient inhibition and interference control processes. So, testing this prediction would require a measure of automaticity, which is not measured as interference effect.^8^

#### Abstractness and/or flexibility of the representation

Cipora et al. (2016) interpreted their results of weaker/non-existent SNARC in adult professional mathematicians by referring to abstractness and or flexibility of their number representations. One of the characteristics of professional mathematicians is the reaching of the formal operation stage as postulated by Piaget. As originally proposed, this is the most advanced stage of cognitive development, allowing abstract thinking and the use of formal logic principles for reasoning. Studies have shown that only about 30% of adults reach this stage (Kuhn et al., 1977).

Being at the stage of formal reasoning may be an important prerequisite for numerical representation to be abstract and flexible. Despite some studies showing that gifted children reach formal operation stage earlier than their peers do (e.g., Carter, & Ormrod, 1982; Keating, 1975), our participants seem to be too young to have fully reached the formal operation stage.

It must also be kept in mind that the professional mathematicians, who were specially trained in mathematics for more than 20 years longer than our sample of gifted children, might have mastered more abstract and flexible number representations (see Butterworth, 2018; Cipora et al., 2016; Sella & Cohen Kadosh, 2018; Siegler & Opfer, 2003). In summary, the gifted children tested in our study most likely have not reached the representation flexibility and abstraction level possessed by professional mathematicians.^9^ This may explain why gifted children did not reveal a weaker SNARC than their peers from the control group (i.e., the effect observed in some adult studies on the SNARC and math skill). Testing whether this was actually the case in case of our participants is not possible, because no such measures were used in the battery. As for now we also do not have a clear idea of a comprehensive measure of representation abstractness and/or flexibility.

### Other observations

Interestingly, in our sample we did not observe a relationship between the SNARC effect and reaction time characteristics. This lack of relationship can be also potentially attributed to the low reliability of the SNARC effect. On the other hand, despite being widely reported in the literature, to the best of our knowledge, this relationship has not been reported in Chinese native speakers. For this reason, it is hard to interpret this result. However, it cannot be attributed to ceiling effects in the performance of Chinese children, as they responded slower and their reaction times were more variable as compared to Western adult samples (see, Cipora et al., 2019a).

### Absence of evidence, evidence of absence, and a gaze into the SNARC file drawer

Given that results of studies reporting (lack of) relationship between the SNARC effect and math skills are quite divergent, one might argue that this is because some studies have found evidence for an effect and some of them simply failed to find such an evidence (either due to power issues, methodological shortcomings, or simply bad luck), which does not preclude that such an effect exist (i.e., absence of evidence for a relationship does not have to imply evidence of absence of a relationship). We believe that this is not the case here and for several reasons, we discuss below.

Firstly, several studies (including the current) utilized Bayesian statistics, which can provide an evidence for absence of the effect in question differentiating it from inconclusive data (which is not possible within frequentist framework). Second, studies not reporting the effect were not characterized by systematically smaller sample sizes than studies revealing the effect. Moreover, low power does not only decrease the probability of observing the effect, which actually exists, but it also increases chances of false positives (e.g., Button et al., 2013). Third, given the file drawer problem and publication bias (it is more likely that positive results are published than null results) we assume that there might be more studies, which did not find effect and were not published, than studies, which found effect and were not published. Please note that in case of several papers lack of relationship between SNARC and math skills was reported as a side finding not being the main objective of the study (e.g., Bonato et al., 2007; Bull et al., 2013). Fourthly, the observation that the direction of relationship between the SNARC effect and math skills differs between children and adults also indirectly suggests that several mechanisms (going in opposite directions) can be at play (some of them more salient in children and some of them more salient in adults), it is also quite likely that the factors cancel each other out. Finally, in several studies both math skills and the SNARC effect were quantified in more than one way, and the results did not vary depending on which method was chosen. This also indirectly suggests that null results in some studies should not be solely attributed to failures to find an existing effect due to incorrect selection of measures of interest.

To sum up, it seems that both discrepancies in the literature and null results reported here cannot easily be accounted for occasional failures to find an evidence for truly existing relationship between the SNARC effect and math skills.

### Limitations and future directions

Despite the general conclusiveness of the presented study, some limitations need to be kept in mind and addressed in future investigations. First, the reliability of the parity judgment task was very low, mostly due to the small number of repetitions of each number in each experimental block. On the other hand, even with four repetitions, SNARC data can be quite stable on a group level (see Nuerk, Iversen, & Willmes, 2004, for stable data with four repetitions per number and hand). Indeed, our data also seem to be quite stable on a group level. In our data preparation process, the trimming eliminated the outlier reaction times, and the values of mean, and in particular the intraindividual variance in RTs were similar to those reported in adult studies (Cipora et al., 2019b). As we discussed in detail before, despite being problematic for correlation analysis, the reliability is not such a problem for between-group comparisons. Importantly, our null results for between-group comparisons held irrespective of the group allocation method.

Second, our controls may be not very representative of China. They were students in the same Beijing school as our gifted children, but they received the normal curriculum. In addition, socioeconomic status (SES) of Bejingers are higher than the average of the whole nation, and SES correlates with mathematical achievement (e.g., Van Ewijk, & Sleegers, 2010 for a review), which perhaps indicates our controls are better in math than average Chinese students. Nevertheless, if we selected the controls from a different school in an economically average city, there might be some other confounds. Additionally, it needs to be mentioned that a robust SNARC effect was observed in the control group, and its size was similar to values reported in other studies testing children of a similar age (− 9.82 in our study, − 11.2 in Schneider et al., 2009; − 13.97 in Bachot et al., 2005; − 11.37 in Georges et al., 2017). Therefore, the SNARC effect in the control group highly resembled the SNARC effect reported in other studies. While we cannot exclude that the control group is not representative of China, the SNARC effect is in the normal range in this group; therefore the available results do not point to major SNARC differences due to sampling.

Representative sampling does not only refer to sample selection within cultures, but also across cultures. For SNARC, like most other cognitive effects, there is a problematic WEIRD (Western, Educated, Industrialized, Rich, Democratic) bias in psychological science, which is known to affect even basic sensory and cognitive effects (e.g., Rad, Martingano, & Ginges, 2018). For the SNARC effect, we know that culture affects the effect (e.g., Shaki, Fischer & Petrusic, 2009). By providing data from normal and highly gifted children in an Eastern culture, we believe that this study can contribute to a more general picture of SNAs. Note for instance, that previous studies illustrated that Chinese children differed from their peers in Western cultures in several aspects of number processing (e.g., automatic processing of numerical magnitude in Stroop-like tasks, Zhou et al., 2007; and the age at which the SNARC effect can be first observed, Yang et al., 2014). As outlined above, the SNARC effect in our control group in China did not differ much from Western children of a similar age—this seems to indicate that cultural attributes do not modulate the SNARC effect on a group level in a major way. However, it also needs to be noted that null mean differences of course do not necessarily imply that we are looking at the same distribution and the same underlying processes. A missing correlation between the SNARC effect and overall RT, which has been found in most studies in WEIRD populations so far can be a hint suggesting such differences. Our study is the first to examine the SNARC effect in highly gifted children and compare them to controls. We found no difference between these groups. Whether this result is generalizable to other cultures, and in particular, the dominant cultures in psychological science and SNARC research, the WEIRD cultures, remains to be shown.

One might argue that the way we operationalized math skills is somehow unclear because we tested gifted children and only measured their arithmetic performance using a timed task. Consequently, one might argue that it is hard to relate the arithmetic performance score to math skills in general. Admittedly, studies investigating links between SNARC and math skills operationalized math skills in very different ways, such as (1) performance on (timed) calculation tasks; (2) standardized math tests (e.g., Kramer, Bressan, & Grassi, 2018); (3) school grades (e.g., Schneider, Grabner, & Paetsch, 2009); (4) self-reported school grades (e.g., Cipora et al., 2019a); and (5) curriculum/field of studies pursued by the participants (e.g., Hoffmann, Mussolin, Martin, & Schiltz, 2014a, b). Therefore, there is a clear discrepancy between studies, and one can hardly think of any consensus here. Some studies have utilized multiple methods (e.g., Cipora & Nuerk, 2013). This is also the case for our study: we used both a timed arithmetic test as well as group allocation. Importantly, gifted children had been allocated to the special curriculum on the basis of their intellectual capabilities before they started the first grade. Therefore, by the time they were tested in our experiment they had been in special training for several (3–5) years. This means that their mathematical knowledge and experience with math problems of varying sorts differed considerably from their peers receiving normal curriculum in virtually all points present in the math curriculum. As our results show, this was clear in the case of the arithmetic task. Nevertheless, future studies should use broader range of math skills measures considering conceptual knowledge, geometry, or algebra. Noteworthy, math skills can probably not be treated as psychologically uniform construct. On the contrary, various cognitive processes (e.g., working memory, conceptual knowledge, or automation of processing) and affective demands (e.g., presence of time pressure) are associated with solving different math tasks. Therefore the *math skills* is rather mathematical (i.e., skills necessary to solve mathematical problems), than psychological category (see Cipora et al., 2018b). Progress in understanding of what constitutes math skills will also help us understand links between different SNAs and different math skills (see also Cipora et al., 2020a).

At the same time, future studies should consider multiple mechanisms on how math skills might relate to the SNARC effect (Cipora et al., 2020b), and the fact that these mechanisms might act in opposite direction. If this is the case, despite the lack of differences in the SNARC effect, groups might still differ in processes driving the SNARC effect (or in relative contributions of these processes). For instance, groups might differ in extent of automatic number processing or in abstractness and/or flexibility of representation. If one of the processes becomes more salient in a given setup or group, one can observe the relationship between the SNARC effect and math skills. This is even more likely if the measure of math skills is tapping on the process, which is most prominent for the formation of the SNARC in this setup (e.g., the automaticity of processing of numbers).

Finally, we wish to make a point, which we have outlined in some previous papers. The SNARC effect, as a directional SNA, is probably not representative for all SNAs (see Cipora et al., 2015, 2018a; Patro et al., 2014, for taxonomies and reviews). Gifted children might outperform controls in extension SNAs, especially in cases in which precise SNA is relevant and helpful in task performance, such as number line estimation (see Hoard et al., 2008 for evidence that intellectually gifted first graders outperformed controls in this task; see also Sella et al., 2016 for similar evidence with professional mathematicians). Therefore, also following our own arguments and taxonomy, we want to make clear that the null difference between gifted children and normal controls is observed here for the most frequently studied SNA, the SNARC effect, but this does not mean or even suggest that there are no differences for other SNAs.

## Conclusion

In conclusion, this was the first study to examine the SNARC effect and its relationship with mathematical / arithmetic skill in gifted children as compared to typically developing controls. We replicated the SNARC effect in a large sample of gifted children. However, there were no significant differences in the SNARC effect between gifted children and the controls, no matter which additional criteria were used for group sampling. The SNARC effect did not correlate with intelligence nor math skills in our sample. These findings are in line with most of previous studies suggesting that the SNARC effect is not related to arithmetic skill and extends these findings within the normal skill range to the highly skilled range of gifted children. However, a lack of between group differences in the observed SNARC effect does not imply that SNAs in general do not differfor other SNAs effects (e.g., number line estimation), a relationship with arithmetic skill/intelligence seems to exist. In the broader sense, the current research is in line with the view that different SNAs must be distinguished and that this distinction is necessary already for children.

## Electronic supplementary material

Below is the link to the electronic supplementary material.

Supplementary file1 (DOCX 22 kb)

## Footnotes

1 We would like to thank an anonymous Reviewer for bringing this point to our attention.

2 On the other hand, a recent meta-analysis shows that fluid intelligence correlates only moderately with math skills – on average r = .41, and the correlation increases with age (Peng, Wang, Wang, & Lin, 2019).

3 When numbers 1,2,3,4,6,7,8,9 (i.e., excluding 0 and 5) are considered, the parity and magnitude predictor are orthogonal. In such a case the slopes for the magnitude predictor (i.e., the SNARC effect) do not differ depending on whether the parity predictor is included or not. Therefore, results of such an analysis are perfectly comparable with studies which do not consider the parity predictor.

4 Formal comparisons reported here consider analysis including 0 and 5. The results are the same when these numbers are excluded.

5 Neither the SNARC nor ST-SNARC for any number range differed between grades, classes, and there was no grade × class interaction. Grade: *F*s ≤ 2.44, *p*s ≥ .091. Class: *F*s ≤ 2.27, *p*s ≥ .134. Grade × class: *F*s ≤ .29, *p*s ≥ *.*746.

6 As there were very few participants in the control group, in an additional analysis we selected the 20^th^ - 80^th^ percentile according to Chinese RSPM norm as controls (*n* = 44), and compared them with gifted children (*n* = 44). Again, there were no differences: the SNARC effect without 0 and 5, *t* (86) = 1.68, *p* = .097, *d* = 0.36, BF_01_ = 1.311; the ST-SNARC effect without 0 and 5, *t* (86) = 0.60, *p* = .550, *d* = 0.13, BF_01_ = 3.829; the SNARC effect with 0 to 9, *t* (86) = 0.93, *p* = .358, *d* = 0.20, BF_01_ = 3.069; the ST-SNARC effect with 0 to 9, *t* (86) = 0.28, *p* = .777, *d* = 0.06, BF_01_ = 4.333.

7 In this analysis there are very few children in the gifted group. Thus, in an additional analysis we selected the top 10% according to the Chinese norm on the arithmetic task as gifted children (*n* = 28), and compared them with the controls (*n* = 47). Again, there were no differences: the SNARC effect without 0 and 5, *t* (73) = 0.57, *p* = .572, *d* = 0.14, BF_01_ = 3.541; the ST-SNARC effect without 0 and 5, *t* (73) = 0.15, *p* = .879, *d* = 0.04, BF_01_ = 4.031; the SNARC effect with 0 to 9, *t* (73) = 0.51, *p* = .613, *d* = 0.12, BF_01_ = 3.641; the ST-SNARC effect with 0 to 9, *t* (73) = 0.06, *p* = .949, *d* = 0.02, BF_01_ = 4.064.

8 However, for exploratory purposes we calculated correlations between SNARC effect measures and the interference index (RT incompatible – RT compatible) in the physical size condition of the numerical Stroop task. Correlations ranged from (-.03 to .03), and none of them was significant. This implies no direct link between automatic number magnitude processing and the SNARC effect (keeping in mind the caveats discussed in the main text).

9 We do not claim that such representational abstractness / flexibility is solely due to practice, but rather that it can be amplified by maturation (as with maturation thinking becomes more abstract in general) and long-term practice. Note that professional mathematicians tested by Cipora et al. (2016) were also much above the population average as regards fluid intelligence, and at least some of them might have been classified as gifted.
