# Supplementary material for: A gifted SNARC? Directional spatial–numerical associations in gifted children with high-level math skills do not differ from controls
Source: Psychol Res. 2020 May 24;85(4):1645–61. doi: 10.1007/s00426-020-01354-9 (PMC8211597; doi:10.1007/s00426-020-01354-9)
Supplement: Supplementary file 1 — Supplementary file1 (DOCX 22 kb) [file 426_2020_1354_MOESM1_ESM.docx]

**Supplementary Material**

**The MARC effect**

When a parity judgment task is utilized, another phenomenon can be observed besides the SNARC effect: odd / even numbers are responded to faster on the left / right hand side. This phenomenon is referred to as the MARC (Markedness Association of Response Codes) effect (Nuerk et al., 2004).

**The MARC effect and between group comparison**. Analysis of the MARC effect parallels the analysis of the SNARC effect reported in the main text (again, more negative slopes correspond to stronger MARC effects). Interestingly, the MARC effect was present neither at the whole sample level nor in any group. Groups also did not differ between each other. Null hypothesis models were also favoured by the Bayesian analyses (cf. Table S1). The results are not surprising because the MARC effect is relatively weak when Arabic numbers are used (Nuerk et al., 2004). Several studies have not found it whatsoever (see e.g., Nuerk et al., 2005; Cipora et al., 2019c). Moreover, it is very heterogeneous between participants (Cipora, Soltanlou, Reips, & Nuerk, 2019a; Cipora, Soltanlou, Smaczny, Göbel, & Nuerk, 2019c; Huber et al., 2015, for the reversed effect in left-handers; see also Krajcsi, Lengyel, & Laczkó, 2018 for evidence for large within- and between-subject variability of the SNARC effect).

Table S1 the Different MARC Effects between Gifted Children and the Controls

| Task property | | | | MARC | | ST-MARC | |
| --- | --- | --- | --- | --- | --- | --- | --- |
|  |  |  |  | 1–4 6–9 | 0–9 | 1–4 6–9 | 0–9 |
| Reliability | | | | 0.79 | 0.71 | 0.70 | 0.70 |
| SD slope | | | | 231.31 | 187.93 | 0.76 | 0.40 |
| % neg. slopes | | | | 0.49 | 0.47 | 0.49 | 0.47 |
| Slope | Overall | mean | | -24.68 | -8.15 | -0.01 | -0.02 |
|  |  | t-test against 0  (*df* = 164) | *t* | -1.37 | -0.56 | -0.25 | -0.3 |
|  |  |  | *p* | .172 | .578 | .806 | .765 |
|  |  |  | *d* | 0.11 | 0.04 | 0.02 | 0.02 |
|  |  |  | BF_01_ | 4.602 | 9.873 | 11.17 | 11.02 |
|  | Gifted | mean | | -13.19 | -7.35 | -0.07 | -0.05 |
|  |  | t-test against 0  (*df* = 73) | *t* | -0.66 | -0.38 | -0.75 | -0.58 |
|  |  |  | *p* | .510 | .707 | .459 | .561 |
|  |  |  | *d* | 0.08 | 0.04 | 0.07 | 0.07 |
|  |  |  | BF_01_ | 6.340 | 7.293 | 5.966 | 6.650 |
|  | Control | mean | | -34.03 | -8.80 | 0.03 | 0.01 |
|  |  | t-test against 0  (*df* = 90) | *t* | -1.2 | -0.41 | 0.35 | 0.18 |
|  |  |  | *p* | .234 | .681 | .727 | .857 |
|  |  |  | *d* | 0.13 | 0.04 | 0.04 | 0.02 |
|  |  |  | BF_01_ | 4.313 | 7.955 | 8.133 | 8.497 |
|  | Group comparison | t-test  (*df* = 163) | *t* | 0.57 | 0.05 | -0.79 | -0.58 |
|  |  |  | *p* | .567 | .961 | .430 | .562 |
|  |  |  | *d* | 0.09 | 0.01 | -0.12 | -0.09 |
|  |  |  | BF_01_ | 5.091 | 5.909 | 4.434 | 5.064 |

**The correlation analysis for the MARC effect and other measures.** All the correlations between the SNARC effect and the MARC effect were not significant, *r*s ≤ .15, *p*s ≥ .050. Apart from this, all other correlations for the MARC effect and the other measures are presented in Table S2. RSPM score correlated with the ST-MARC without 0 and 5, *r* = -.16, *p* < .05, BF_01_ = 1.337, and the ST-MARC with 0 to 9, *r* = -.18, *p* < .05, BF_01_ = 0.800, respectively. However, Bayesian evidence for this correlation are highly inconclusive.

Table S2 the Correlations between the MARC effect and the other measures

| Variable | (1) | (2) | (3) | (4) | (5) | (6) | (7) | (8) |
| --- | --- | --- | --- | --- | --- | --- | --- | --- |
| (1) MARC  (1-4 6-9) | — | <.001 | <.001 | <.001 | 1.335 | 0.669 | 9.194 | 6.759 |
| (2) ST-MARC (1-4 6-9) | .83^***^  (.78; .87) | — | <.001 | 0.001 | 6.890 | 6.265 | 1.337 | 7.398 |
| (3) MARC  (0-9) | .84^***^  (.79; .88) | .84^***^  (.79; .88) | — | <.001 | 1.252 | 0.122 | 3.423 | 8.006 |
| (4) ST-MARC (0-9) | .75^***^  (.67; .81) | .94^***^  (.92; .96) | .89^***^  (.85; .91) | — | 7.779 | 5.514 | 0.800 | 7.187 |
| (5) Mean RT | -.16  (-.30; -.01) | -.07  (-.22; .08) | -.16  (-.31; -.01) | -.06  (-.21; .10) | — | <.001 | 0.004 | <.001 |
| (6) SD (RT) | -.18  (-.33; -.03) | -.08  (-.23; .08) | -.23  (-.37; -.08) | -.09  (-.24; .07) | .88^***^  (.84; .91) | — | 0.023 | <.001 |
| (7) RSPM score | -.03  (-.189; .13) | -.16^*^  (-.31; -.00) | -.12  (-.27; .04) | -.18^*^  (-.33; -.02) | -.31^***^  (-.45; -.16) | -.28^***^  (-.42; -.13) | — | <.001 |
| (8)Arithmetic score | -.07  (-.23; .09) | -.06  (-.22; .09) | -.05  (-.21; .10) | -.07  (-.22; .09) | -.39^***^  (-.25; -.52) | -.37^***^  (-.23; -.50) | .35^***^  (.20; .48) | — |

Note: Below the diagonal: Pearson correlations (95%CI); *p < .05, **p < .01, ***p < .001; Above the diagonal: BF_01_.

**Summary.** The evidence of the MARC effect, its correlates and developmental trajectories in the literature is much scarcer than for the SNARC effect. Nevertheless, our results at least partly correspond to those of Berch et al. (1999), who found the MARC effect in sixth graders (mean age 11 years 8 months; range 8 years 2 months - 12 years 7 months) but not in third and fourth graders (age range 8 years 4 months - 10 years 8 months). Additionally, the MARC effect has not always been found in experiments using Arabic numbers, while it was more pronounced when number words or auditory numbers were used (Nuerk et al., 2004). We are also not aware of any study reporting the MARC effect in native Chinese speakers. Thus, we refrain from making strong conclusions about the MARC effect here.

**Gender differences in the SNARC and MARC effects**

The SNARC effect, and not the MARC effect, was robust both in girls and boys. There were no gender differences in either the SNARC effect or the MARC effect (Table S3).

Table S3 Gender Differences in both the SNARC Effect and the MARC Effect

| Group | Measure |  | SNARC | | ST-SNARC | | MARC | | ST-MARC | |
| --- | --- | --- | --- | --- | --- | --- | --- | --- | --- | --- |
|  |  |  | 1–4 6–9 | 0–9 | 1–4 6–9 | 0–9 | 1–4 6–9 | 0–9 | 1–4 6–9 | 0–9 |
| Girls | mean |  | -9.63 | -7.94 | -0.22 | -0.21 | -9.48 | 3.87 | 0.02 | 0.01 |
|  | t-test against 0 (*df* = 72) | *t* | -3.89 | -3.59 | -4.07 | -4.17 | -0.38 | 0.18 | 0.19 | 0.07 |
|  |  | *p* | < .001 | < .001 | < .001 | < .001 | .705 | .859 | .847 | .944 |
|  |  | *d* | 0.46 | 0.42 | 0.48 | 0.49 | 0.04 | 0.02 | 0.02 | 0.01 |
|  |  | BF_01_ | 0.010 | 0.025 | 0.006 | 0.004 | 7.246 | 7.648 | 7.634 | 7.750 |
| Boys | mean |  | -6.28 | -6.77 | -0.16 | -0.18 | -36.74 | -17.69 | -0.04 | -0.03 |
|  | t-test against 0 (*df* = 91) | *t* | -2.67 | -3.33 | -3.68 | -4.46 | -1.44 | -0.89 | -0.56 | -0.52 |
|  |  | *p* | .009 | .001 | < .001 | < .001 | .154 | .374 | .576 | .602 |
|  |  | *d* | 0.28 | 0.35 | 0.38 | 0.47 | 0.15 | 0.09 | 0.06 | 0.05 |
|  |  | BF_01_ | 0.309 | 0.054 | 0.019 | 0.001 | 3.205 | 5.916 | 7.453 | 7.610 |
| Group comparison | t-test  (*df* = 163) | *t* | 0.97 | 0.39 | 1.48 | 0.85 | -0.75, | -0.73, | -0.5 | -0.37 |
|  |  | *p* | .331 | .697 | .142 | .394 | .454 | .466 | .617 | .711 |
|  |  | *d* | -0.15 | -0.06 | -0.23 | -0.13 | 0.12 | 0.11 | 0.08 | 0.06 |
|  |  | BF_01_ | 3.826 | 5.507 | 2.154 | 4.232 | 4.556 | 4.619 | 5.264 | 5.546 |
